# Development of an Integrating Sphere-Based Wide-Range Light Source System for the Linearity Evaluation of a Photodetector Used in Radiation Detection and Bioanalysis Instruments

**DOI:** 10.3390/s24237544

**Published:** 2024-11-26

**Authors:** Tetsuro Matsumoto, Akihiko Masuda, Minoru Tanabe, Seiya Manabe, Hideki Harano, Kazuki Niwa

**Affiliations:** National Metrology Institute of Japan, National Institute of Advanced Industrial Science and Technology, AIST Central, 1-1-1 Umezono, Tsukuba 305-8568, Japan; t-matsumoto@aist.go.jp (T.M.); aki-masuda@aist.go.jp (A.M.); tanabe-m@aist.go.jp (M.T.); s-manabe@aist.go.jp (S.M.); h-harano@aist.go.jp (H.H.)

**Keywords:** BNCT, photodetector, linearity, integrating spheres, light source

## Abstract

We developed a compact wide-range light source system for evaluating the linearity of photomultiplier tube (PMT) output. This system utilizes two integrating spheres equipped with a continuously variable slit and output aperture to modulate a stabilized light-emitting diode light source, producing an output light range as wide as seven orders of magnitude. To verify the wide linearity range of the integrating sphere system, three silicon photodiodes coupled with electric current readers monitored the light intensity and simultaneously confirmed each other’s linearity. Using this system, we evaluated the linearity of the PMT used in a neutron detector we are currently developing and found it to have a linear range of more than four orders of magnitude. Non-linearity characteristics were also successfully measured in detail at a higher output range. Neutron detector operation requires both calibration of the detection efficiency and evaluation of the linearity between the neutron dose and its output. These results indicate that this system is a simple and useful method to evaluate the linearity of photodetectors used in radiation detectors and other applications.

## 1. Introduction

**Figure 1:**
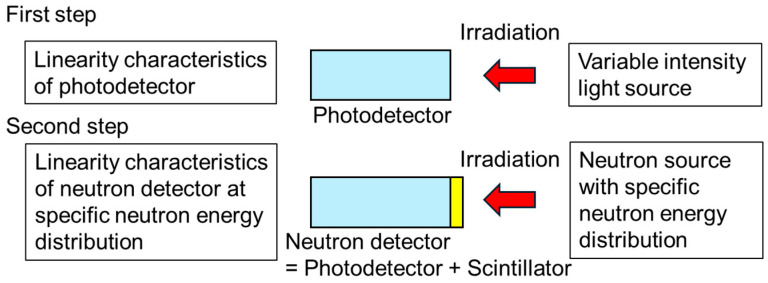
Our concept for the evaluation of the linearity of a neutron detector. Blue shows a photodetector, and yellow shows a scintillator.

Photodetectors, including photomultiplier tubes (PMTs), silicon photodiodes (SiPDs) and silicon photomultipliers, are widely used in analytical instruments across various fields, which is also true for scintillation-based neutron detectors [1,2,3,4]. Due to the growing demand for the accurate evaluation of high-intensity neutrons used in boron neutron capture therapy (BNCT), various types of detectors have been developed, including semiconductor detectors and proportional counters with neutron-sensitive gas. The detection efficiencies of neutron detectors used in BNCT facilities are determined through calibrations in reference neutron fields [5,6,7]. The neutron fluence rate in a typical reference neutron field is less than 10^5^ cm^−2^ s^−1^ in most cases, depending on conditions, whereas a neutron fluence rate of over 10^9^ cm^−2^ s^−1^ is used in BNCT facilities [8,9]. We are also developing a neutron detector composed of a ^6^Li glass scintillator and a PMT [1]. It is possible to measure high-intensity neutrons used in BNCT facilities using current outputs from the anode of a PMT. The neutron detector should be also capable of measuring the neutron intensity of reference neutron fields. To indicate that the neutron detector calibrated in the reference neutron field can precisely measure neutrons at BNCT facilities, it is also necessary to evaluate linearity characteristics to determine the relationship between the output from the neutron detector and the incident neutron fluence rate, which has a range of more than four orders of magnitude. The first candidate evaluation method for the linearity characteristics of neutron detectors is to directly measure neutrons with different intensities using radionuclide neutron sources, accelerator-based neutron sources, and reactor-based neutron sources. However, it is difficult to fine-tune the incident neutron fluence rate using direct neutron methods. Furthermore, no two neutron sources have a perfectly identical neutron spectrum. Results obtained with the direct neutron method have very discrete data [10]. Radiation detectors that use photodetectors, such as scintillator-type radiation detectors, have an upper limit, because photodetectors have an upper limit to their outputs. They are also known to deviate from linearity near the upper limit, especially for PMTs [11]. With the direct neutron method, it is impossible to obtain information on slight deviations from linearity. Therefore, it is impossible to precisely determine the application range of a neutron detector for the neutron fluence rate or neutron dose. Thus, we propose to evaluate the linearity of a neutron detector using the concept described in Figure 1, which targets a neutron detector comprising a scintillator and a photodetector, because the linearity of a scintillator-based neutron detector depends on the linearity of the photodetector for incident light. First, the linearity of a photodetector used in a neutron detector is evaluated using a variable-intensity light source. Next, after attaching a scintillator to the evaluated photodetector, its detection efficiency is calibrated using a neutron source with a specific neutron energy distribution and a certain intensity in a reference neutron field. Finally, the linearity of the neutron detector is obtained using data on the photodetector’s linearity and calibration data in the reference neutron field.

Many linearity evaluation methods for photodetectors have been studied [12,13]. A flux addition method (or superposition method) is often used because it is able to precisely evaluate linearity, although the equipment tends to be large and complicated. Furthermore, it takes time and effort when there are many evaluation devices. We aim not only for the linear evaluation of photodetectors, but also for the simplicity of the method using a compact light source. For this purpose, we developed an integrating sphere-based system equipped with a light-emitting diode (LED) light source and SiPDs. The goal is then to have a variable incident light intensity with a range of more than four orders of magnitude for a photodetector using this light source system. In this paper, we describe the details of the newly developed system, report the tests for the same type of PMT used in a neutron detector, and discuss the results.

## 2. Integrating Sphere-Based Light Source System

**Figure 2:**
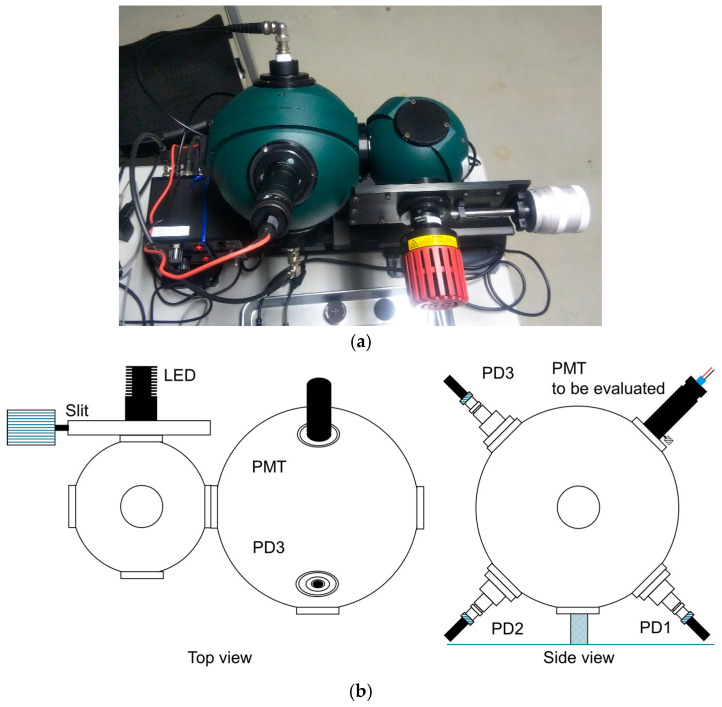
(**a**) Photo and (**b**) top and side schematic views of the integrating spheres based on a wide-range light source system for the linearity evaluation of a photodetector.

Figure 2 shows a photo and schematic view of an integrating sphere-based wide-range light source system for the linearity evaluation of a photodetector. The system consists of 6-inch and 4-inch integrating spheres (Labsphere, North Sutton, NH, USA, SPH-6 and SPH-4, respectively), three SiPDs (Hamamatsu Co., Shizuoka, Japan, S2281), and an LED light source with a 565 nm central wavelength (Thorlabs M565L3, Newton, NJ, USA). The inner wall of the integrating sphere is coated with barium sulfoxide as a reflective material, and the light introduced through the input port repeats diffusive reflection inside. The LED light source is set on the 4-inch integrating sphere, and the SiPDs and PMT to be evaluated are set on the 6-inch integrating sphere. Baffles were placed between the ports on both integrating spheres to prevent direct light incidence. A shutter was set in front of the LED light source to allow the detector settings to be changed while the light source was turned on. A manual slit attenuator (Labsphere, North Sutton, NH, USA, VAM-010, made of aluminum) was also provided between the 4-inch integrating sphere and the LED light source to allow adjustment of the light intensity. The SiPDs and PMT were set on a plane perpendicular to the axis connecting the centers of the 4-inch and 6-inch integrating spheres. Therefore, the SiPDs and PMT provided an incident light source under the same conditions.

An integrating sphere is an optical component that enables photodetectors at several ports on the sphere to make equivalent measurements simultaneously. Light is introduced through a port from any light source, such as LEDs or incandescent lamps. In the present study, a stabilized LED is used because its wavelength range can be limited. The brightness of LEDs can be adjusted electrically, but this is undesirable here because the temperature of the element changes and the spectrum also changes. Instead, the use of a slit attenuator allows the light from the LED to be spatially attenuated while maintaining the spectrum at the light input port. The attenuated ratio of the light introduced into the sphere is continuous and in proportion to the area of the attenuator.

The SiPDs with independently verified linearities monitor the light intensity inside the integrating sphere. The three SiPDs were confirmed to be linear in a range of more than five orders of magnitude by measurement using a superposition method with a laser device [12]. However, the reliable linear dynamic range of the SiPD is also restricted by the instrumentation, including the I/V amplifier and the digital multimeter (DMM) that reads the electrical signal output from the SiPD, which also needs to be checked separately. Therefore, three SiPDs were employed to verify the response linearity mutually. Here, it is essential that the three SiPDs always provide comparable light measurements irrespective of the incident light intensity, and the integrating sphere is the optical device best suited to make this possible. However, the integrating sphere, which makes light uniform within its interior, also has imperfections, in particular the mechanical movement of the slit that controls the incident light intensity, which can impair the optical equivalence among the three SiPDs. Coupling the two spheres is expected to increase the uniformity of the spatial distribution of light inside the 6-inch integrating sphere in the system and ensure optical equivalence among the SiPDs. By using such an integrating sphere, light with an accurately evaluated attenuation rate can exit the output port. The intensity range of the output light can be varied by attaching another aperture with a diameter of 2 μm, 10 μm, 50 μm, 200 μm, or 1 mm (Thorlabs P2K, P10K, P50K, P200K, and P1000K, Newton, NJ, USA) to the output port for attenuation and by geometrical arrangement. The aperture has a black oxide coating foil of 300 series stainless steel. In this system, the SiPDs consist of a main PD1 without an aperture and a PD2 and PD3 with apertures having diameters of 1 mm and 10 mm for validation. This allowed the linearity for the PMT between the upper and lower limits to be verified. In front of the PMT to be evaluated, an aperture with a diameter from 2 μm to 1000 μm was installed to adjust the amount of light incident on the PMT. The aperture in front of the PMT was able to change the range, whereas the slit allowed for fine adjustment.

## 3. Measurements

**Figure 3:**
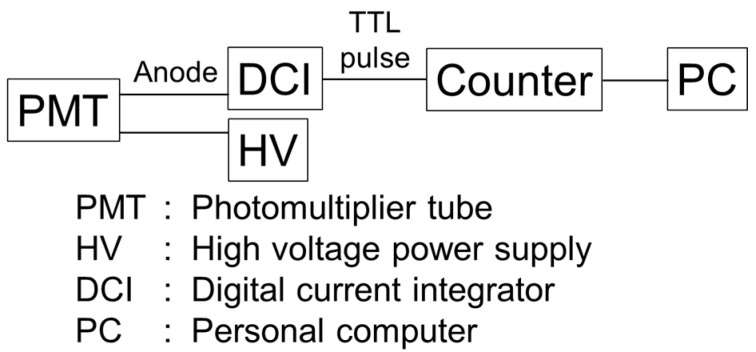
Block diagram of the electronics for the photomultiplier tube (PMT) to be evaluated.

In the present study, a PMT (Hamamatsu Co., Shizuoka, Japan, H3165-10 [14]) with a 10 mm-diameter photocathode area of the same type as that used in the current mode neutron detector in our previous study [1] was used in the linearity test with the integrating sphere-based wide-range light source system. Figure 3 shows a block diagram of the electronics for the PMT evaluated in the present study.

Output currents were taken from the anode of the PMT assembly. The output current was detected by a digital current integrator (DCI, AMETEK ORTEC 439, Oak Ridge, TN, USA), and the output pulses from the DCI were counted by a LabVIEW (Version 2024 Q1 64-bit) counter (NI USB-6341, Austin, TX, USA). A gate time of 1 s was set in these measurements as the exposure time. The number of pulses accumulated in the gate time was stored in a personal computer. In some BNCT facilities, pulsed neutron beams are often used with an accelerator [15]. In these cases, it is desirable to measure current with an ammeter with a fast time constant or a charge meter. The latter was employed in the present study. The DCI outputs single transistor–transistor logic (TTL) pulses for a constant charge and has three modes: 10^−10^ C/pulse, 10^−8^ C/pulse, and 10^−6^ C/pulse [16]. To confirm consistency among the modes, the data overlap in each of them was compared. In the PMT specifications, the recommended applied voltage between the anode and cathode is −1000 V, with a maximum of −1250 V. In neutron measurements, applied voltages lower than the specifications are often used. Therefore, in the present study, data were obtained for applied voltages of −700 V, −800 V, −900 V, −1000 V, and −1200 V.

The outputs of the three SiPDs used as monitors were read with the same gate time as the PMT by a DMM (Agilent, Santa Clara, CA, USA, 34401A) through an I/V amplifier (FEMTO Messtechnik GmbH, Berlin, Germany, DLPCA-200 for PD1 and Hamamatsu Co., Shizuoka, Japan, C9329-01 for PD2 and PD3). The data were imported into a personal computer through a GPIB bus.

For each high voltage applied to the PMT, aperture in front of the PMT, and DCI mode, the slit in front of the LED light source was manually opened. Measurements were repeated at least 10 times for each condition of the continuously variable slit. Naturally, the number of repetitions increased as the output current decreased. In particular, when the output from the PMT was less than 1 nA, the output current was integrated to at least 100 nC. The uncertainty for each repetition measurement was less than 0.01%, indicating the stability of this system. Before the measurement, the background current, which consists of the dark current, was measured. Table 1 shows the dark currents obtained for each applied voltage and their standard deviations. The results in Table 1 are the average dark currents obtained when apertures of 2 μm, 10 μm, 50 μm, and 200 μm were installed. The leakage of light from the outside world into the integrating sphere must be considered as a possible background. The output current due to light leakage is sufficiently smaller than the dark current, due to adequate light shielding for the system. Table 1 shows that the dark current increased as the applied voltage increased from −700 V to −1200 V, and that the dark current increased rapidly at the voltage recommended by the manufacturer.

In the linearity evaluation using this system, we used the factor *G* obtained with the following formula:

(1) $$G=\frac{I_{PMT}}{S\times I_{PD}}$$

where *S* (cm^2^) is the area of the aperture attached to the front of the PMT and *I_PD_* (A) is the output current of the SiPD used as a monitor. In the case of this system, it was obtained as a voltage value proportional to the output current, due to the use of the I/V amplifier. *I_PMT_* (A) is the output current of the PMT to be evaluated after subtracting the background. In Equation (1), *S* × *I_PD_* can be expressed as the relative incident light intensity. If *I_PMT_* has linearity for a specific aperture, *G* should be constant and dependent on *S*.

## 4. Results and Discussion

### 4.1. Results for PMT Linearity Tests

**Figure 4:**
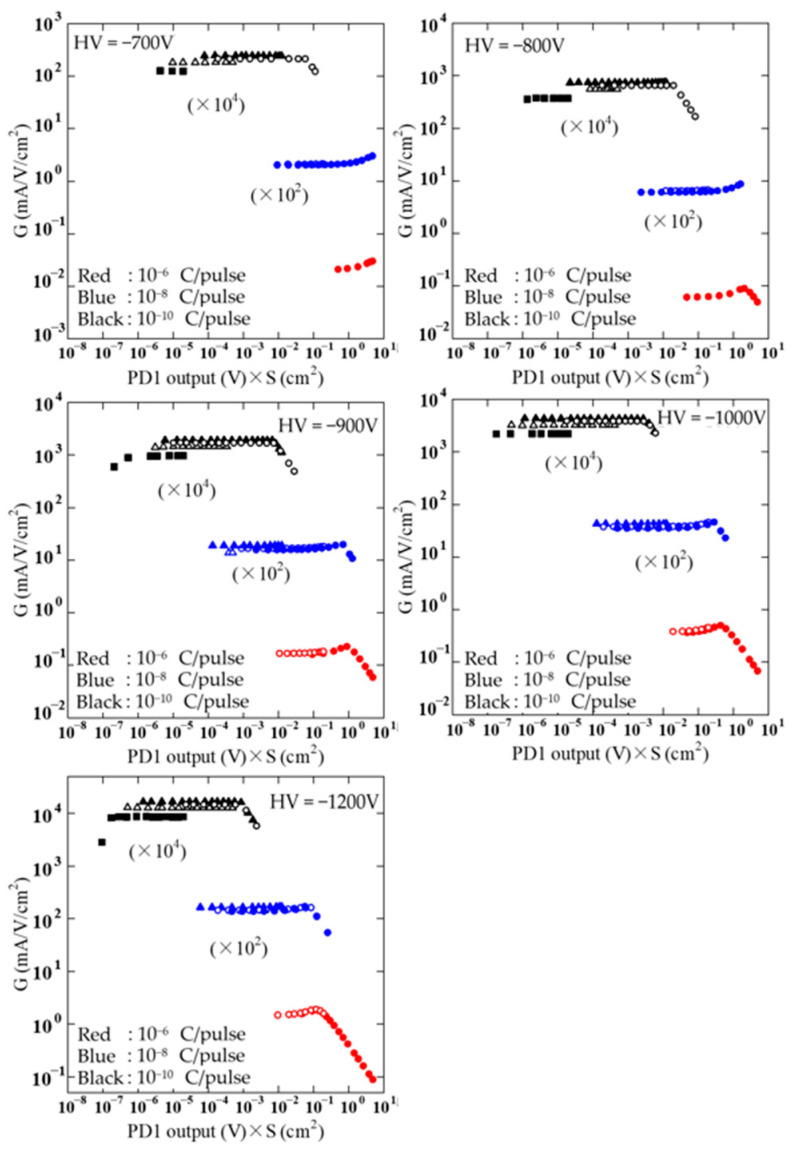
Raw experimental results of the linearity characteristics of the PMT with an applied voltage from −700 to −1200 V. The results shown in red, blue, and black correspond to 10^−6^, 10^−8^, and 10^−10^ C/pulse modes in the digital current integrator, respectively. Circles, white circles, triangles, white triangles, and squares are the results for apertures of 1000 μm, 200 μm, 50 μm, 10 μm, and 2 μm, respectively.

**Figure 5:**
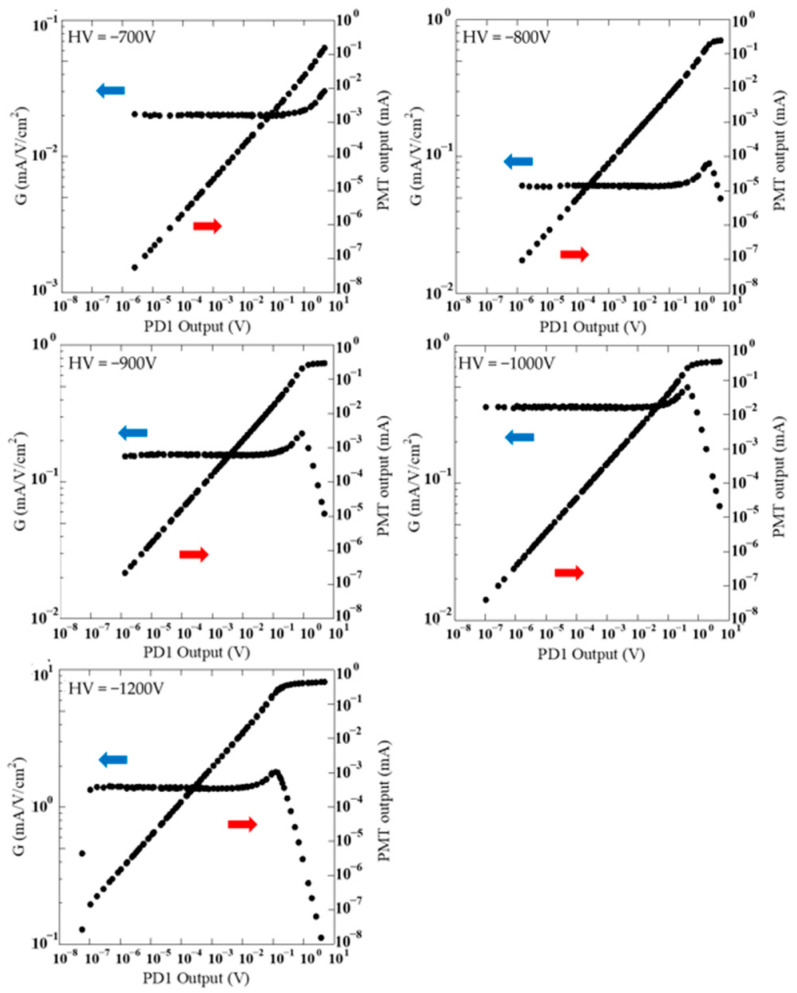
Corrected experimental results of the linearity characteristics of the PMT under applied voltages from −700 to −1200 V.

Figure 4 shows the results of the linearity experiments for voltages from −700 V to −1200 V applied to the PMT. The output of PD1 is used for the horizontal axis in Figure 4. The reason why the *G* value becomes small near the maximum incident light intensity relative to the 10^−6^ and 10^−8^ C/pulse mode data is because the limit of the DCI pulse outputs has been reached. The results were obtained using the area of the physical nominal in the specification as the aperture area. Figure 4 indicates that the plots are not consistent for different apertures. In general, when the aperture is placed in front of a photodetector, the amount of light incident on the photodetector is not proportional to the physical nominal area of the aperture [17]. In these experiments, the light incident on the PMT from the integrating sphere had various angles, resulting in a difference in light intensity between the center and the edge of the aperture. From the results shown in Figure 4, the relationship between *G* values for the apertures in each measurement was obtained as shown in Table 2. In Table 2, experimental results were extracted for each aperture condition at first where *G* is flat within 1%. The results are the ratio of *G* averaged over the range of flatness between apertures. The results are not listed in Table 2 for the 10^−6^ C/pulse mode in the DCI, because there was no range of flatness under its condition. The “Average” in Table 2 shows the average values of results obtained with applied voltages from −700 V to −1200 V. For each voltage, the effect of aperture area agreed within 0.8%. To set the PMT in this system, it was inserted into the socket and fixed by a screw. There is a possibility of slight PMT installation position deviation. It is considered that the uncertainty of the effect of aperture area is related to the reproducibility of the PMT position. It is also possible that the stability of the LED light source caused the uncertainty. Although an evaluation of the effective aperture area is beyond the scope of this paper, repeated measurements using this system will allow for a more detailed evaluation. Figure 5 shows the experimental results corrected using the effective aperture area shown in Table 2. In Figure 5, data resulting from saturation above the upper limit of the DCI output have been removed. First, Figure 5 shows that this light source can smoothly vary light intensity over a range of more than seven orders of magnitude. This was achieved by simply replacing the aperture and opening and closing the slit installed in front of the light source. It is possible to easily evaluate linearity over an extremely wide range.

### 4.2. Verification of Reference SiPD

**Figure 6:**
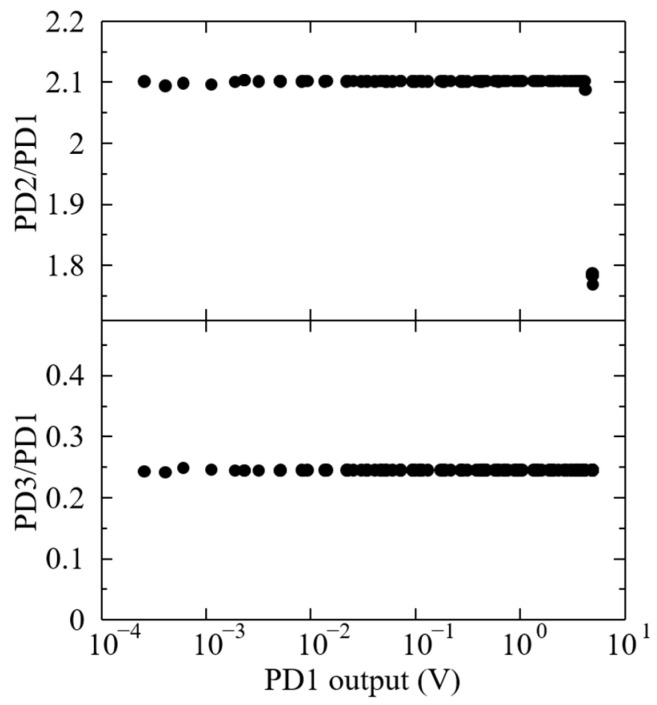
Results for PD2/PD1 and PD3/PD1 ratios with respect to PD1 in experiments when −900 V was applied.

Although the output of PD1 was used for the horizontal axis of the results shown in Figure 4 and Figure 5, it is necessary to ensure that the output range of PD1 is in the range where linearity is ensured. Pedestals used for the I/V amplifier and DMM were also taken into account for PD1, PD2, and PD3, because they could influence the photodetector linearity. Therefore, we compared the outputs between PD1 and PD2, and between PD1 and PD3, as shown in Figure 6. In these results, the background due to dark current was subtracted. Figure 6 shows that the relationship between the outputs of PD1 and PD2 and between PD1 and PD3 are constant within 0.1%. The results indicate that soundness around the upper and lower limits of the applicable range of PD1 was verified by PD2 and PD3, because the sensitivity difference appears to depend on the geometric arrangement in the integrating sphere. The sudden decrease in the PD2/PD1 result around the PD1 output of 5 is because the readable range of the DMM for the output of PD2 has an upper limit. These results indicate that the linearity soundness of the photodetector consisting of the I/V amplifier, DMM, and PD1 was validated. Furthermore, the results also indicate the stability of the system.

### 4.3. PMT Linearity Characteristics

The PMT tested in the present study deviated from linearity as the amount of incident light increased, and the output current did not increase thereafter. This is a typical characteristic of PMTs [11]. When the linearity was assumed to exist in a range where *G* is flat within 1%, the upper and lower limits of linearity for each condition were obtained as shown in Table 3. Linearity for the PMT was successfully confirmed in the range of more than four orders of magnitude for all conditions. Both the upper and lower limits tend to increase as the applied voltage increases from −700 V to −1000 V. However, the tendency breaks down below −1000 V, which is the manufacturer’s recommended voltage. If the lower limit is about 10 times the background, the lower limit of linearity may be obtained up to a much lower output when −700 V and −800 V are applied. However, at −700 V and −800 V, the measurement time became very long for the lower output range. Thus, measurement using the integrating sphere-based light source will need to be automated.

In the future, by attaching a ^6^Li glass scintillator to this PMT and performing measurements in a reference neutron field, the results in Figure 5 can be used to evaluate its linearity as a neutron detector. In such cases, it would be possible to evaluate the linearity by applying exactly the same principle by using an integrating sphere larger than the one used in this experiment. It was also clearly observed that the PMT characteristics were different depending on the applied voltage. This is important information for the future use of PMTs in neutron detectors for BNCT.

### 4.4. Application

In radiation measurements, PMTs with 2-inch and 3-inch diameters are also often used. The integrating sphere wide-range light source system allows for evaluation in any spectra by attaching an appropriate light source to the input port. Also, any detectors can be attached to the output port, enabling the evaluation of a variety of detectors. Furthermore, by guiding the light from the output port using an optical fiber cable, this system is suitable for photodetection devices that are difficult to fix directly on the port. Examples include multi-channel spectrometers and even larger detectors.

Another advantageous application of this wide-range light source system is the dynamic range evaluation of light measurement devices, which are widely applied in the field of bioanalysis, due to the high reliability of the output light intensity ratio over a wide range of up to seven orders of magnitude. Especially in the fields of biotechnology, the food industry, life science, and medicine, this wide-range light source system is very advantageous when applied to the instruments used in most analytical methods [18]. In these fields, analytical methods utilize optical signal measurements, including bioluminescence, chemiluminescence, and fluorescence signals. In many cases, the optical signal values from samples are compared by their ratios. Here, it is essential that the optical signal measurements be made in the linear dynamic range. Furthermore, color discrimination techniques are applied to extract more information from test samples using multiple stains, such as for the classification or subtype discrimination of cancer [19,20]. For color discrimination, the ratio of detected signals is used, in which the complete linearity of the detector is also essential [21]. For this reason, ISO 24421, established in 2023 [22], recommends that the dynamic range of bioanalysis instruments be evaluated. Typical users of bioanalysis instruments have used a dilution series of samples as a linear light source, but this task is not easy outside of laboratories where biological samples are available. Unforeseen errors can also occur during sample preparation. The integrating sphere-based wide-range light source system proposed in this report is able to evaluate the linear dynamic range of the optical signal measurement instruments independently from using biological samples. Thus, the integrating sphere system can be widely applied to the linearity evaluation of photodetectors other than those used in radiation detectors and the light sources that are detected using them.

In this system, the linearity of the reference SiPD was evaluated in advance by means of the flux addition method [12]. However, blind reliance on the assumed linearity could cause unforeseen errors, potentially undermining the reliability of the entire system. In addition, the reliability of the reference SiPD must be confirmed as a part of the photodetector setup, including the I/V amplifier and DMM. To verify the linearity of the reference SiPD as a complete photodetector set, three sets of photodetectors were evaluated through intercomparison, ensuring the reliability of this system. However, this intercomparison also comes with drawbacks, namely increased measurement time and more complicated data analysis. To address this, it would be worthwhile in future work to introduce a flux addition method to this system. Such self-calibration methods can directly assess the linearity of photodetectors, including both SiPDs and PMTs, and are expected to broaden the applications of this system. This issue should be pursued alongside the automation of measurements.

## 5. Conclusions

To evaluate the linearity of scintillator-based neutron detectors with respect to the neutron fluence rate, a compact wide-range light source system based on integrating spheres was developed. The system was composed of an LED light source and SiPDs whose linearities were evaluated by the flux addition method as a monitor. Experiments successfully demonstrated that measurements of the PMT to be evaluated can smoothly vary light intensity over a range of more than seven orders of magnitude by simply replacing the aperture and the continuously variable slit installed in front of the LED light source. In this way, the exact linearity characteristics of the PMT were precisely evaluated. This system can be applied to all measurement devices that use photodetectors. This will directly lead to the evaluation of the linearity of the neutron detector by attaching a ^6^Li glass scintillator to the PMT and testing it in a reference neutron field. This will enable a detailed linearity evaluation of a neutron detector that is not possible using neutrons. Thus, this system would be effective for improving the measurement accuracy of dose assessment for high-intensity neutrons at BNCT facilities. Photodetectors, including PMTs and SiPDs, are widely used in analytical instruments across various fields, indicating that this simple and practical system can be applied to a wide range of instruments.

## Figures and Tables

**Table 1:** Background current for each voltage applied to the photomultiplier tube (PMT).

Voltage (V)	Current (nA)	Standard Deviation (nA)
−700	6.19 × 10^−4^	5.40 × 10^−4^
−800	1.08 × 10^−3^	1.25 × 10^−3^
−900	1.54 × 10^−2^	9.05 × 10^−3^
−1000 *	1.88 × 10^−2^	1.84 × 10^−3^
−1200	8.10 × 10^−2^	8.90 × 10^−3^

* Recommended voltage by the manufacturer.

**Table 2:** Ratio of the average *G* over the range of flatness between apertures under each voltage condition and pulse mode. 200/1000, 50/1000, 50/200, 10/200, and 2/200 are the ratios of the results for 200-μm and 1000-μm apertures, 50-μm and 1000-μm apertures, 50-μm and 200-μm apertures, 10-μm and 200-μm apertures, and 2-μm and 200-μm apertures, respectively. “Average” means the average of results obtained under conditions from −700 V to −1200 V. Numbers in parentheses under the average values are standard deviations.

**−700 V**				**−1000 V**			
**10^−8^ C/Pulse**		**10^−10^ C/Pulse**		**10^−8^ C/Pulse**		**10^−10^ C/Pulse**	
200/1000	0.97	50/200	0.87	200/1000	0.94	50/200	0.89
		10/200	1.15	50/1000	0.82	10/200	1.18
		2/200	1.71			2/200	1.75
**−800 V**				**−1200 V**			
**10^−8^ C/Pulse**		**10^−10^ C/Pulse**		**10^−8^ C/Pulse**		**10^−10^ C/Pulse**	
200/1000	0.93	50/200	0.87	200/1000	0.96	50/200	0.87
50/1000	0.82	10/200	1.15	50/1000	0.83	10/200	1.14
		2/200	1.766			2/200	1.71
**−900 V**				**Average**			
**10^−8^ C/Pulse**		**10^−10^ C/Pulse**		**10^−8^ C/Pulse**		**10^−10^ C/Pulse**	
200/1000	0.94	50/200	0.88	200/1000	0.95 (0.77%)	50/200	0.87 (0.51%)
50/1000	0.82	10/200	1.16	50/1000	0.83 (0.45%)	10/200	1.16 (0.59%)
		2/200	1.76			2/200	1.74 (0.69%)

**Table 3:** Upper and lower limits of the linear region of the PMT outputs obtained by the integrating sphere system.

Condition (V)	Upper Limit (mA)	Lower Limit (mA)
−700	4.45 × 10^−3^	8.84 × 10^−8^
−800	4.23 × 10^−2^	1.50 × 10^−7^
−900	1.14 × 10^−2^	5.47 × 10^−7^
−1000	1.03 × 10^−2^	6.35 × 10^−8^
−1200	1.46 × 10^−2^	4.05 × 10^−7^

## Data Availability

The data are contained within the article.
